# A techno-practical method for overcoming the biotoxicity and volatility obstacles of butanol and butyric acid during whole-cell catalysis by *Gluconobacter oxydans*

**DOI:** 10.1186/s13068-020-01741-9

**Published:** 2020-06-03

**Authors:** Xia Hua, GenLai Du, Xin Zhou, Ali Nawaz, Ikram ul Haq, Yong Xu

**Affiliations:** 1 Key Laboratory of Forestry Genetics & Biotechnology (Nanjing Forestry University), Ministry of Education, Nanjing, 210037 People’s Republic of China; 2 Jiangsu Co-Innovation Center of Efficient Processing and Utilization of Forest Resources, College of Chemical Engineering, Nanjing Forestry University, No. 159 Longpan Road, Nanjing, 210037 People’s Republic of China; 3 Jiangsu Province Key Laboratory of Green Biomass-based Fuels and Chemicals, Nanjing, 210037 People’s Republic of China; 4 Institute of Industrial Biotechnology, GC University, Lahore, 54000 Pakistan

**Keywords:** Butyric acid, Butanol, *Gluconobacter oxydans*, Whole-cell catalysis, SOS bioreactor, Energy co-factor

## Abstract

**Background:**

Butyric acid is a platform chemical material, the production of which has been greatly stimulated by the diverse range of downstream applications in many industries. In particular, higher quality butyric acid used in food and medicine, is more dependent on microbiological production methods. Hence, the bio-oxidation of butanol to butyric acid has been identified as a promising method with good potential economic and environmental benefits. However, both butanol and butyric acid are usually intensively toxic to most microorganisms as well as the bio-oxidation pathway. To develop a green, efficient and competitive microbiological method is the primary work to overcome the bottleneck of butyric acid industry.

**Result:**

A combined bioprocess was designed with alternative whole-cell catalysis for butyric acid bio-conversion from butanol by *Gluconobacter oxydans* in a sealed-oxygen supply bioreactor (SOS). In the operation system, the escape of volatile substrates and toxic chemicals to cells can be avoided by the use of a sealed bioreactor, combined with the rejuvenation of cells by supplying energy co-factors. Finally, during a one-batch whole-cell catalysis, the utilization rate of substrate increased from 56.6 to 96.0% by the simple skill. Additionally, the techno-practical bioprocess can realize the purpose of cell-recycling technology through the rejuvenation effect of co-factor. Finally, we obtained 135.3 g/L butyric acid and 216.7 g/L sorbose during a 60-h whole-cell catalysis. This techno-practical technology provides a promising approach to promote the industrial production of butyric acid with more competitiveness.

**Conclusion:**

The techno-practical biotechnology has powerfully promoted the process of butyric acid production by microorganisms, especially makes up for the lack of aerobic fermentation in the industry, and surmounts the shortcomings of traditional anaerobic fermentation. At the same time, this technically practical system provides a promising approach for the promotion of the industrial production of butyric acid in a more competitive manner.

## Background

Butyric acid (BA) is a short chain fatty acid with the chemical formula CH_3_–CH_2_–CH_2_–COOH with the industrial price of $2200/t (butanol $800/t). BA is an important synthetic raw material which has been widely used in the field of food [1], medicine [2], feed [3] and agriculture [4]. With the expansion of the market scale in recent years and the increasing variety of downstream products, the global demand for BA is also expanding rapidly. Currently, the main butyric acid production methods are chemical and microbial methods [5]. According to different materials, chemical methods are mainly divided into 3 categories: alkane oxidation, aldehyde oxidation and alcohol oxidation [6]. Alkane oxidation methods cannot support large-scale industrial production because of the requirement for expensive catalysts and environmental hazards. Moreover, the aldehyde oxidation method uses butyraldehyde as the substrate, which is even more expensive than BA, making it difficult to obtain any economic benefits in industrial applications. As for alcohol oxidation, although the yield of BA is satisfactory and conforms to the requirements of environmentally friendly development, the interference of alkali will inevitably cause equipment corrosion. In addition, the microbiological method uses monosaccharides such as glucose as a substrate for anaerobic fermentation to prepare BA using different microorganisms [7, 8]. However, there are some limitations to the production of BA by anaerobic fermentation, such as low productivity and the complexity of the metabolic pathway, resulting in a BA yield using anaerobic fermentation of less than 50% [9–12]. Compared with chemical methods, microbiological methods lack a competitive economical advantage [8]. For example, Liu et al. used *C. tyrobutyricum* mutant PAK-EM to produce BA from glucose via batch-fed anaerobic fermentation, which obtained 43 g/L of BA with the yield of 0.5 g/g [13]. Therefore, to develop a techno-practical bioprocess for BA preparation is essential to solve the limitations of product yield and production costs using the current microbiological methods.

Currently, the bottleneck of traditional anaerobic fermentation for BA production has not been solved effectively. Therefore, it is difficult to make a breakthrough in the development of the BA industry by improving anaerobic fermentation biotechnologies alone. Moreover, BA production by the biotechnology of aerobic fermentation is restricted many factors which have not been definitely reported. Firstly, most aerobic microorganisms realize complete oxidation, so the final product is not stopped at BA. Secondly, aerobic fermentation is also limited by the lack of economic competitiveness, which is reflected by poor product yields and productivity. Therefore, the industrial production of BA by microbiological methods has not been realized worldwide. However, with the long-term high oil prices and consequential impact on operational costs, bioenergy plays an increasingly important role in future production. Meanwhile, BA preparation by microbiological methods will increasingly exhibit its unique advantages with advancement of current research [14]. In addition to the considerable market prospect of BA, large-scale production of BA by microbiological methods is a key development trend in the future.

Industrial production of BA by microbiological methods mainly relies on identifying suitable microorganisms to meet the basic industrial requirements. Fortunately, bacterial strain *Gluconobacter oxydans* (*G. oxydans*), as a representative of Gram-negative bacteria, is a promising microbe for bio-converting biomass-based butanol to BA. Nowadays, many studies about the basic mechanisms and application of *G. oxydans* have been reported [15–17]. One of the most advantageous characteristics of *G. oxydans* is that it has a series of membrane-bound dehydrogenases on the surface of cell membranes, including alcohol dehydrogenase, aldehyde dehydrogenase, glycerin dehydrogenase and glucose dehydrogenase [18, 19]. Moreover, the membrane-bound dehydrogenases only undergo simple dehydrogenation or oxidation reactions on the cell membrane, allowing *G. oxydans* to convert the substrate into products and release products directly into the periplasm, which greatly improves the catalytic efficiency [20]. In the biotransformation process, metabolizable carbon sources, such as sugars, will enter the cytoplasm through the cell membrane for metabolic reactions, while other substances will only undergo incomplete oxidation reactions on the membrane surface. In general, *G. oxydans* has the advantages of incomplete oxidation and efficient catalysis because of its unique characteristics. In consequence of the outstanding advantages, *G. oxydans* has been widely employed for the industrial production of 1,3-dihydroxyacetone [21], gluconic/xylonic acid [22], sorbose, furoic acid and other high added-value products [23].

The membrane-bound alcohol dehydrogenase of *G. oxydans* can also conduct whole-cell catalysis for the conversion of biomass-based butanol to BA, although to date several studies have utilized this strain to produce BA. The whole-cell catalysis process is based on biomass-based butanol, which is a raw chemical material with a wide range of sources. In 2012, the world production capacity of biomass-based butanol was about 4.1 million tons/year, while the market demand was only 3.2 million tons/year. Obviously, the low demand of the downstream industry leads to the inventory pressure of biomass-based butanol. Hence, research into and development of BA preparation from biomass-based butanol can effectively alleviate the depression of biomass-based butanol stocks. However, due to the aerobic demand of *G. oxydans* in the whole-cell catalysis process, the volatility of biomass-based butanol lead to large losses under ventilated conditions. Moreover, biomass-based butanol and BA easily shuttle through cell membranes due to their small molecular size, which have toxic effects on cells. All these obstacles hinder the development of whole-cell catalysis by *G. oxydans* for BA production. Hence, the primary objective of this study was to develop a targeted and techno-practical bioprocess for BA production by *G. oxydans*.

## Results and discussion

### The whole-cell catalysis of volatile biomass-based butanol in AS-BR

**Fig. 1:**
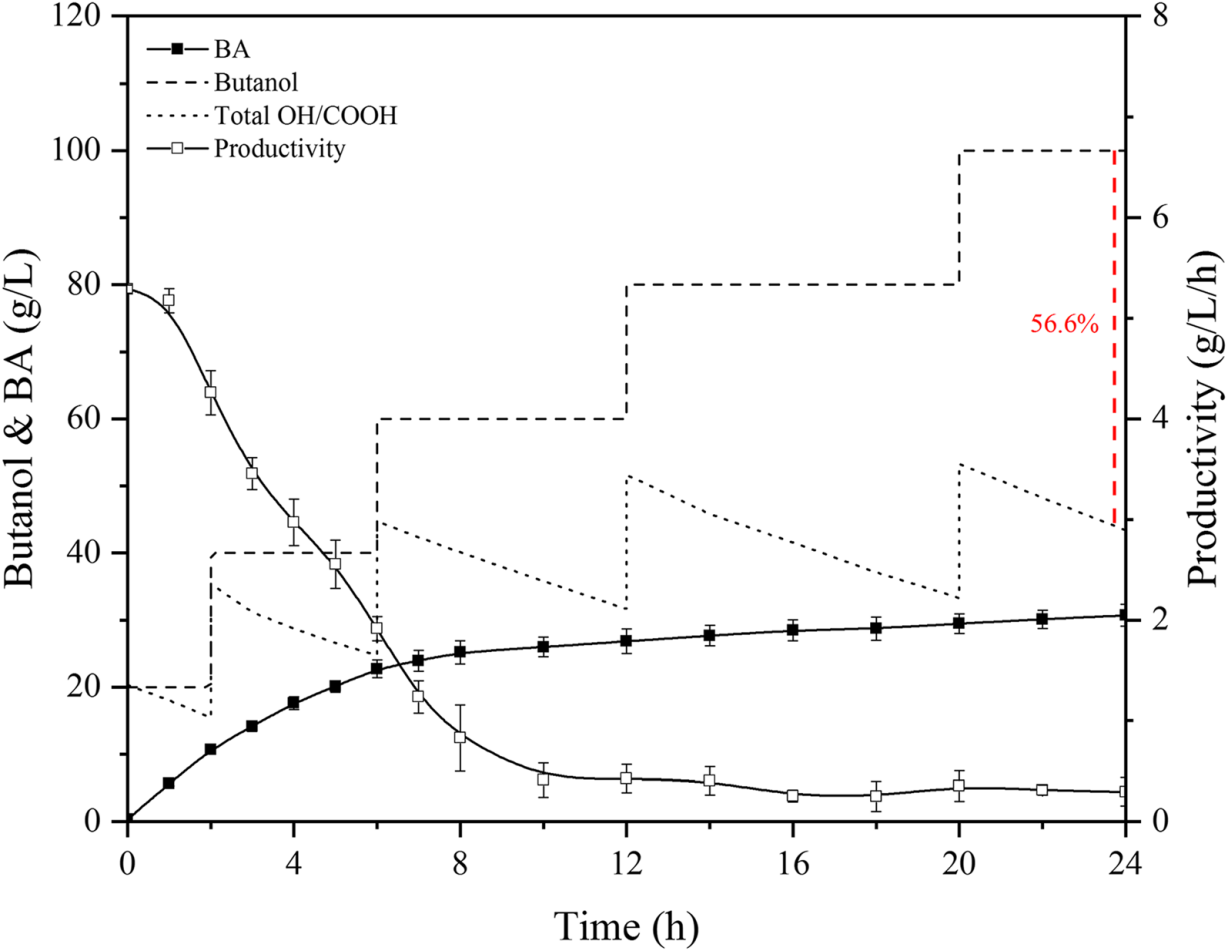
The whole-cell catalysis for BA bioproduction in AS-BR (500 rpm, 3 vvm)

*Gluconobacter oxydans*, one of representative obligate aerobic bacterium, usually uses oxygen as a final electron acceptor for driving a series of oxidation reactions [24]. Hence, maintaining the air flow in the bioreactor is a precondition to ensure *G. oxydans* catalytic efficiency. Conventional bioreactors rely on an air compressor to continuously provide fresh air to meet the aerobic needs of microorganisms. Based on the fact, the whole-cell catalysis was conducted in an AS-BR by *G. oxydans*. As shown in Fig. 1, the substrate biomass-based butanol was batch fed into the bioreactor due to its negative effect of toxicity and substrate inhibition. During 24-h whole-cell catalysis, batch feeding operations were performed whenever substrates were exhausted, 100 g/L biomass-based butanol (5 rounds) were added into the bioreactor. After 24 h, a total production of 30.7 g/L BA and a productivity rate of 1.3 g/L/h were achieved, generating a yield of 35.3%. However, with the accumulation of BA, the productivity decreased gradually due to the negative feedback effect of product inhibition. Particularly, when the catalysis duration reached 8 h, BA accumulation had reached about 25 g/L, with the productivity basically at the lowest level, which can only be maintained at 0.3–0.4 g/L/h. Moreover, curiously, the total concentration of biomass-based butanol and BA after catalysis process was only 43.4 g/L. It is not difficult to find that that under these conditions that *G. oxydans* was unable to metabolize biomass-based butanol for growth and the whole reaction did not comply with the principle of mass balance. Compared with 100 g/L substrate addition, the material was seriously lost by 56.6% in terms of carbon flux. Moreover, the remaining 8.1% butanol was retained in the broth without reaction. In view of this phenomenon, it may be speculated that a large amount of biomass-based butanol was volatilized due to continuous ventilation.

**Fig. 2:**
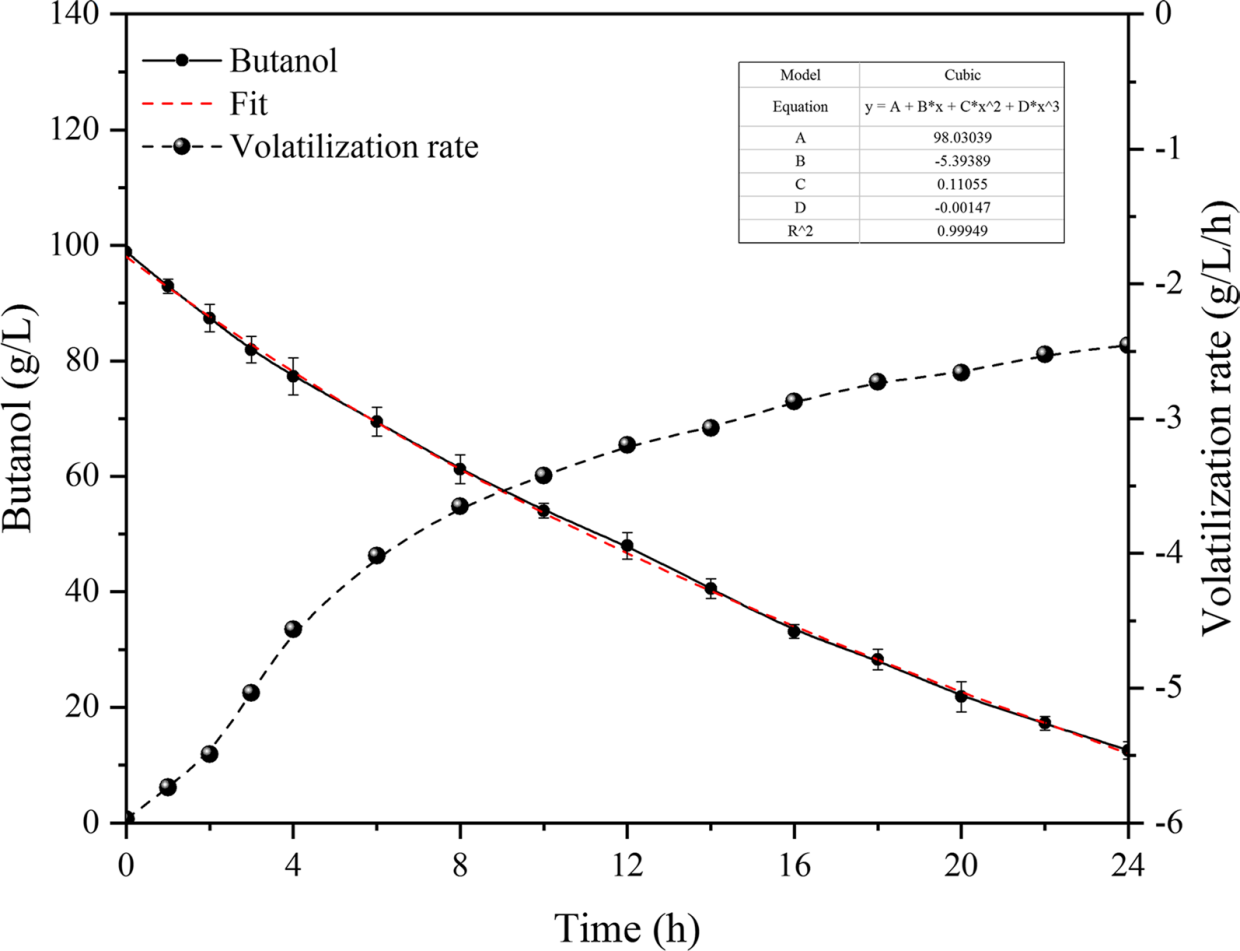
The concentration of biomass-based butanol in AS-BR (500 rpm, 3 vvm)

Based on the phenomenon of mass balance in the bioreactor, biomass-based butanol concentrations were monitored under the same conditions in an AS-BR without the addition of cells. As shown in Fig. 2, the curve represents the kinetic change in biomass-based butanol concentration at conditions of 3 vvm and 500 rpm. It was not surprising that the biomass-based butanol concentration decreased rapidly in the bioreactor without other clear sources of consumption, which decreased from 98.9 to 12.5 g/L in 24 h. According to the curve fitting, fitted by using Eq. (1) and *R*^2^ = 0.999, as shown:

1 $$ y = {\text{A}} + {\text{BX}} + {\text{CX}}^{2} + {\text{DX}}^{3}. $$

The volatilization rate slows down with the decrease in concentration. Since the whole-cell catalysis was performed by batch feeding, 20 g/L substrate was added in each batch. Corresponding to the curve of concentration and volatilization rate, when the substrate was 20 g/L, the biomass-based butanol volatilization rate was 2.5–2.7 g/L per hour under the conditions defined for the AS-BR, which is equivalent to 12.5–13.5% loss of substrate per hour. The loss seriously affected the catalytic efficiency and product yield. Hence, to solve the problem of biomass-based butanol volatilization is a precondition to improving the economic competitiveness of the whole-cell catalysis.

### The whole-catalysis of biomass-based butanol in SOS-BR

Due to the ventilation in the aerobic catalytic system, the escape of volatile matter is inevitable. Simultaneously, the escape of biomass-based butanol is also one of the obstacles to promote the production of BA by aerobic microorganisms. The prerequisite for the feasibility of preparing BA using aerobic microorganisms is to surmount the problem of biomass-based butanol volatilization. Because *G. oxydans* is unable to metabolize biomass-based butanol for growth, it means that the whole-cell catalysis process occurs without any CO_2_ being produced [25]. Based on the fact, we designed an SOS-BR which can achieve bioreactor sealing and pressurization. Moreover, SOS-BR biotechnology also allows the automatic balance of oxygen. When the pressure in the bioreactor increased to 0.05 MPa, the oxygen supply would be stopped automatically, while otherwise, it was automatically supplied with oxygen. A sealed air supplied bioreactor was not employed here mainly because there was less gas present in the sealed system and oxygen in the air was not sufficient to support the oxygen demand of cells as the final electron acceptor. Therefore, we conducted whole-cell catalysis employing sealed operation in the SOS-BR to observe whether the embarrassing situation of biomass-based butanol volatilization could be effectively mitigated.

**Fig. 3:**
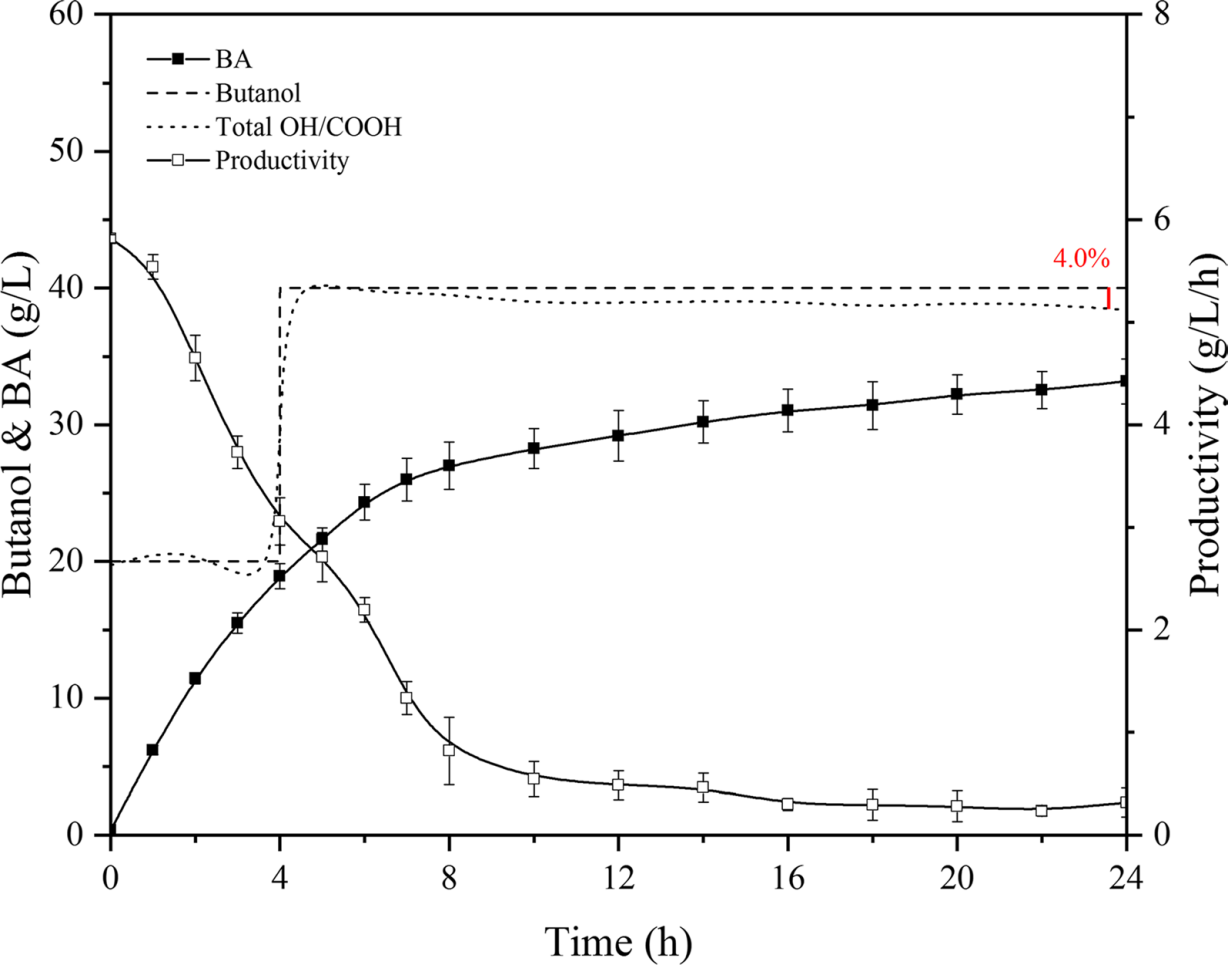
The whole-cell catalysis for BA bioproduction in SOS-BR (500 rpm, 0.01–0.02 MPa)

**Fig. 4:**
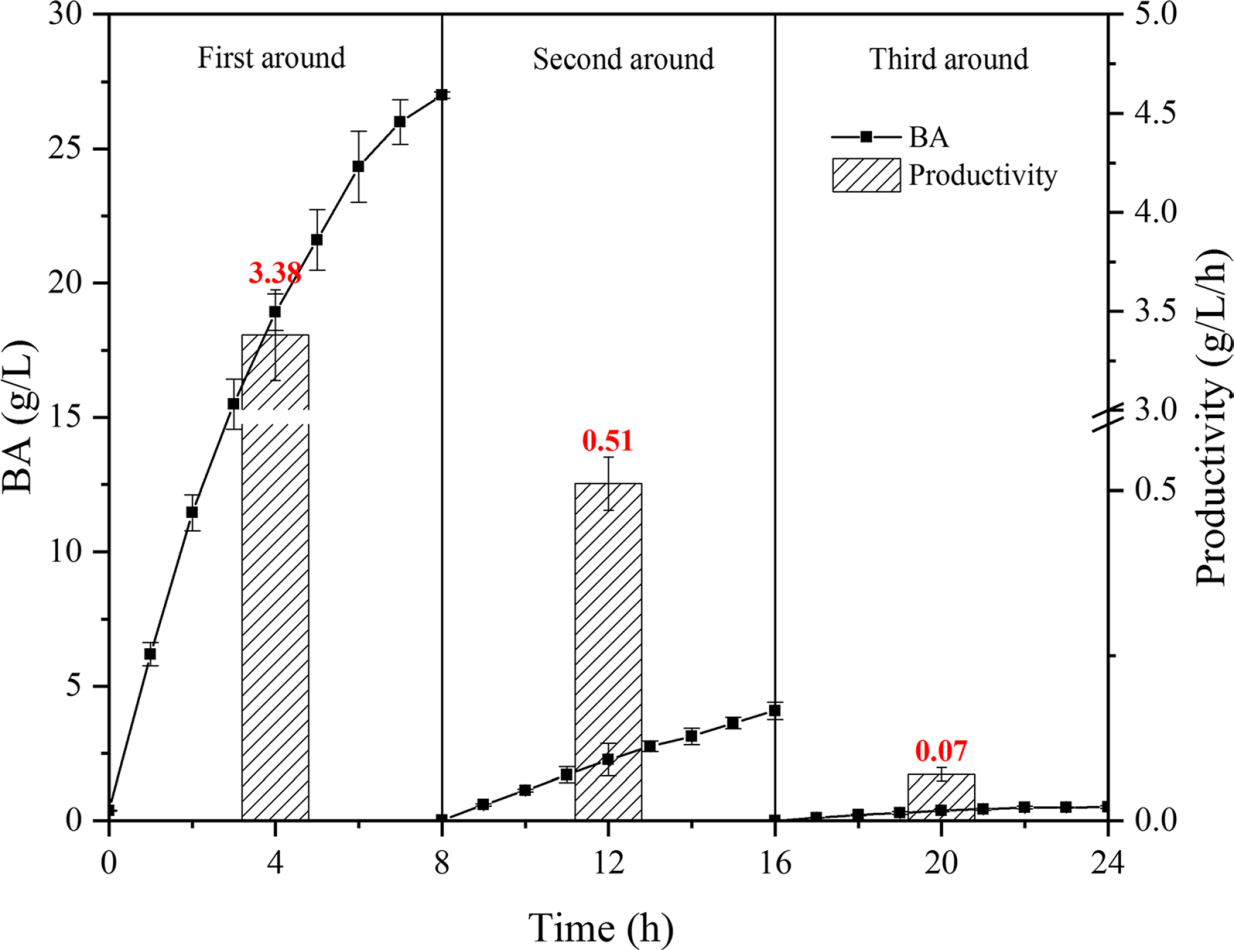
The cell-recycling technology for BA bioproduction in SOS-BR

As shown in Fig. 3, the whole-cell catalysis in SOS-BR with biomass-based butanol as substrate was distinctly presented. The whole process in the SOS-BR was similar to that in the AS-BR, generating 33.2 g/L BA with an average productivity of 1.4 g/L/h. Importantly, only 4.0% of biomass-based butanol was lost in the entire reaction, presumably due to minor volatilization during sampling. Obviously, SOS-BR can completely avoid the issue of biomass-based butanol volatilization and escape, which has far-reaching significance for improving the economic competitiveness of BA preparation by aerobic organisms. However, similarly to AS-BR, the productivity of BA decreased rapidly to 0.3 g/L/h at 8 h, which is an unacceptable level for industrial application. The process presented a typical product inhibition negative feedback effect, with the traditional solution being to adopt cell-recycling technology to conquer the inhibition. Therefore, in the present study cell-recycling technology was applied in order to improve production and productivity. Each round of BA production and productivity is shown in Fig. 4. After 8 h of whole-cell catalysis, the cells were poisoned by biomass-based butanol and BA, making them unrecyclable. In the first 3 rounds of the cell-recycling experiment, the production of BA was 27.0 g/L, 4.1 g/L and 0.5 g/L, respectively. It can be seen that the efficiency of the second round was only 15.2% of that of first round, while the third round exhibited almost no catalytic performance. However, the recycling of cells is a basic requirement of the microbial industry, and hence, realizing the technology purpose of cell-recycling was the core direction of subsequent improvement of the whole-cell catalysis.

### Improvement of cell-recycling technology by co-factor rejuvenation

Previous experiments in this study have shown that cells cannot achieve effective recycling, which seriously restricts the industrialization of BA. In the whole-cell catalysis, small molecules of biomass-based butanol and BA may shuttle through the cell membrane, causing certain toxicity to cells. Therefore, based on the fact, in-depth development of technologies for cell-recycling in BA production is the last key procedure to establishing the feasibility of industrial production of BA by aerobic microorganisms.

**Fig. 5:**
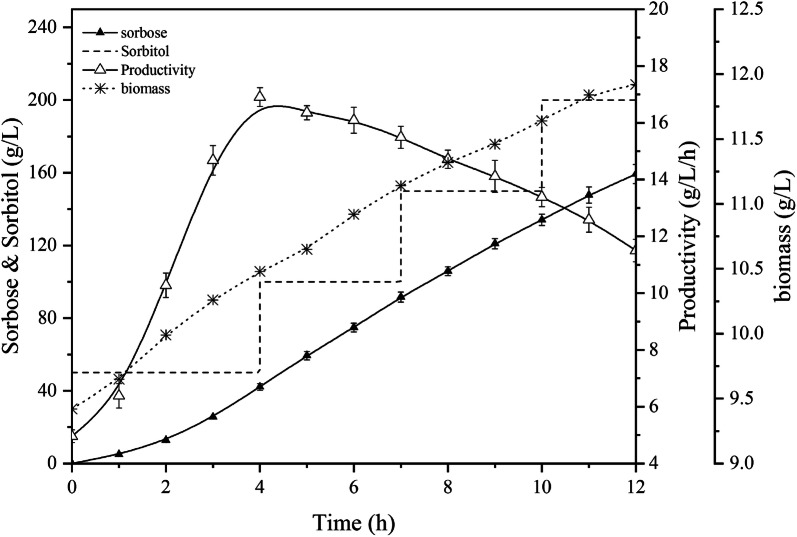
The bioprocess for *G. oxydans* rejuvenation by energy co-factor sorbitol

Whether the cells lose their catalytic activity or die after poisoning is the premise of whether cells can recover their recyclability by special means. If *G. oxydans* becomes dormant due to inhibition by toxicity, the cell-recycling technology remains possible. Sorbitol is the optimal carbon source for *G. oxydans* and most suitable energy co-factor for metabolism. Consequently, sorbitol was employed as a co-factor to rejuvenate *G. oxydans* for 12 h, with the results shown in Fig. 5. From the perspective of production, 159.2 g/L of sorbose accumulated within 12 h with an average productivity of 13.3 g/L/h. However, the initial volume productivity was at an unqualified level and increased from 4.9 to 16.9 g/L/h in 4 h. The subsequent decrease in productivity was due to the inhibition of sorbose and the increase of broth viscosity. Apparently, the incompetence of initial productivity indicated that the cells were inhibited by toxicity due to high concentrations of biomass-based butanol and BA, causing them to enter a semi-dormant state and could not guarantee normal catalytic activity. Fortunately, the rejuvenation effect of sorbitol on cells was distinct. More importantly, during the rejuvenation process, most sorbitol was oxidized to the by-product sorbose, with only 5% of sorbitol metabolized for cell growth, which did not affect the cost of BA production. The process could not only completely recover the physiological activity of *G. oxydans* within 4 h, but also induced a proliferation effect, which could make up for the loss of cells in the operation of centrifugation.

**Fig. 6:**
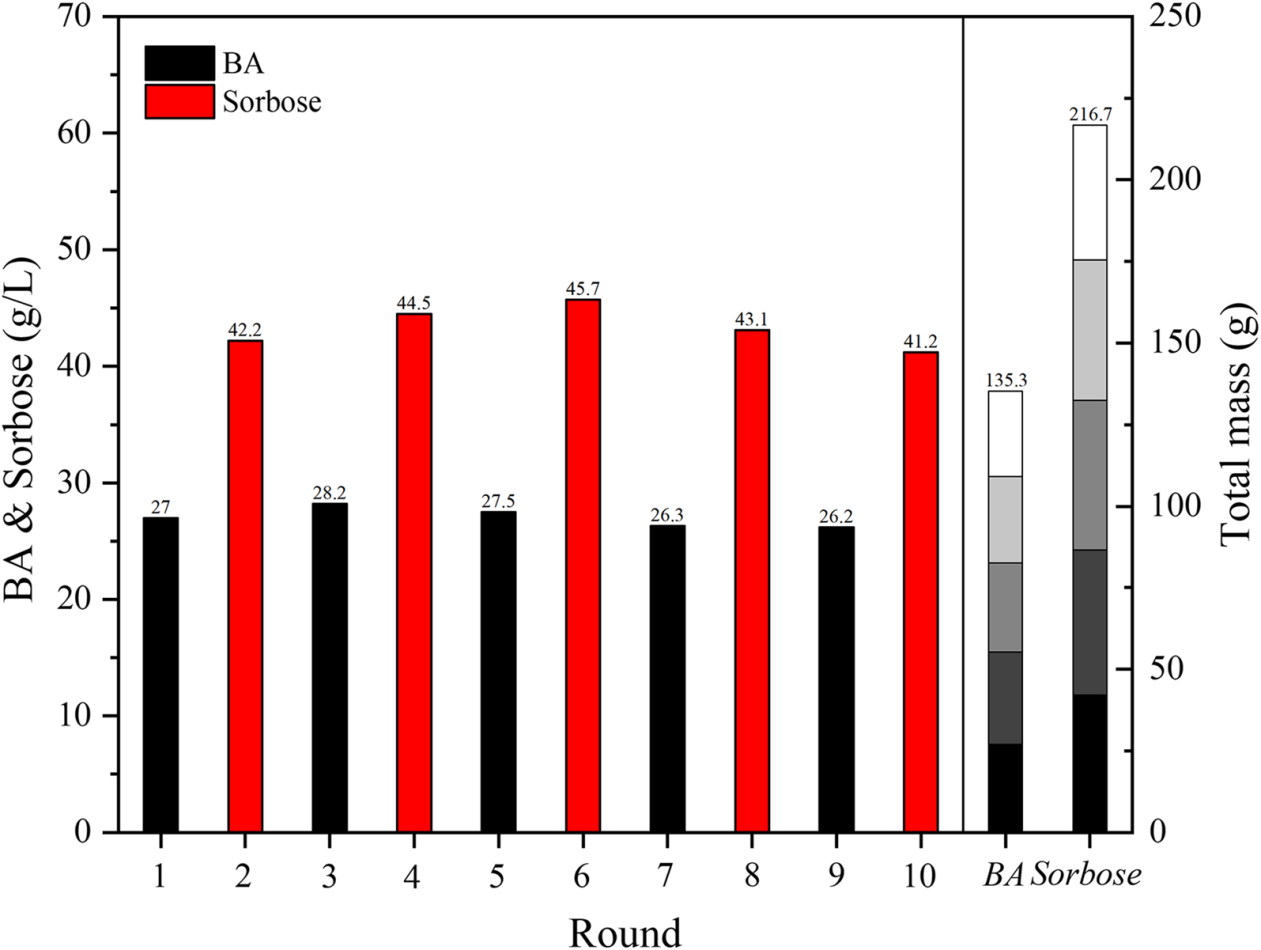
The BA and sorbose concentration in each and total cell-recycle rounds in techno-practical bioprocess

According to the performance of the results of the rejuvenation experiments, the co-factor sorbitol can restore normal physiological function in *G. oxydans* within 4 h. Moreover, the productivity of BA bioproduction by whole-cell catalysis in SOS-BR was at an extremely poor standard at 8 h. Hence, alternative whole-cell catalysis was performed including 8 h of BA preparation and 4 h of rejuvenation. On the basis of ensuring the catalytic performance of *G. oxydans*, alternative catalysis could also produce high concentrations of sorbose, which would not affect the economic competitiveness of BA production and instead bring greater economic benefits. The results of alternative catalysis are clearly shown in Fig. 6. After 10 rounds of cell-recycling experiments, including 5 rounds of whole-cell catalysis and 5 rounds of the rejuvenation process, the catalytic performance of *G. oxydans* did not decline significantly. Finally, 135.3 g/L of BA and 216.7 g/L of sorbose were obtained during 60 h, with a yield of 95% for both. The alternative catalysis process effectively overcame the bottleneck of cell-recycling, producing the additional product sorbose, which greatly strengthened the economic competitiveness of the BA aerobic microbial method.

From the perspective of technical economy, we have made a detailed comparison between the existing microbial fermentation for BA preparation and the techno-practical method, and the results are shown in Table 1. The traditional typical microbial production of BA is an anaerobic fermentation with sugar as substrate and *C. tyrobutyricum*/*C. butyricum* as strain. Apparently, regardless of which sugars were employed as substrates, the BA yield was no more than 50%, and the productivity was not competitive with chemical method. However, with SOS-BR as the reaction vessel, *G. oxydans* as the catalyst, through co-factor rejuvenation and regeneration operation, the integrated process can effectively biocatalytic biomass-based butanol to BA, importantly, the BA yield was up to 95%. Moreover, taking the production of BA by anaerobic fermentation with glucose as a typical case, and assuming the productivity of BA as 100%, the techno-practical bioprocess can greatly increase 283.1%, which excellently improved the economic value of BA production by microbial method.

**Table 1:** The comparison of integrated process and typical anaerobic fermentation for BA production

Strain	Substrate	Time (h)	Production (g/L)	Productivity (g/L/h)	Yield (%)	Comparison (%)	References
*C. tyrobutyricum*	Glucose	74	44.0	0.6	38	–	Zhang et al. [26]
*C. tyrobutyricum*	Glucose	120	43.0	0.4	47	39.3	Liu et al. [13]
*C. tyrobutyricum*	Sugarcane molasses	–	34.6	0.6	–	− 1.6	Jiang et al. [27]
*C. tyrobutyricum*	Flour hydrolysate	50	62.8	1.3	45	+ 111.9	Fayolle et al. [28]
*C. butyricum*	Whey	42	18.6	0.4	39	− 25.4	Vandak et al. [29]
*C. butyricum*	Saccharose	30	7.3	0.2	24	− 59.3	Zigova et al. [30]
*G. oxydans* (*AS*-*BR*)	Butanol	24	30.7	1.3	35	+ 116.9	–
*G. oxydans* (*SOS*-*BR*)	Butanol	24	33.2	1.4	96	+ 133.9	–
*G. oxydans* (*Integrated process*)	Butanol	60	*135.3*	*2.3*	*95*	+ *283.1*	**–**

Where time represents bioreactor tank operation time and the productivity is calculated based on 1-L fermentation broth. During comparison, the productivity of BA production from glucose by *C. tyrobutyricum* is set as 1

## Conclusion

The limitations of BA production by microbiological hinder the development of BA application in food and medical-grade industries. Particularly, in the absence of promising solution for BA preparation by anaerobic fermentation, the breakthrough in preparation using aerobic microorganisms is extremely valuable. Based on the analysis of economic competitiveness, the defect of BA yields caused by biomass-based butanol escape is effectively mitigated by employing SOS-BR. More importantly, the rejuvenation by co-factor flawlessly overcame the limitation that *G. oxydans* cannot be recycled in whole-cell catalysis. Generally, this techno-practical technology provides a potentially feasible approach for the BA bioproduction at an industrial scale.

## Materials and methods

### Microorganism and materials

*Gluconobacter oxydans* NL71 strain, derived from ATCC621, was stored in sorbitol-agar medium containing 50 g/L sorbitol, 5 g/L yeast extract, and 15 g/L agar under 4 °C. *G. oxydans* NL71 inoculum was cultivated in a 250-mL Erlenmeyer flask containing 50 mL of culture medium consisting of 100 g/L sorbitol and 10 g/L yeast extract, then incubated for 24–36 h at 30 °C and 220 rpm. The proliferative cells were harvested by refrigerated centrifugation (Avanti J26 XP, Beckman Coulter) at 6000 rpm for 5 to 10 min [31, 32].

Biomass-based butanol, BA, sorbitol and sorbose were purchased from Macklin, while yeast extract was obtained from Sigma. All other chemicals such as nutrients and NaOH, were of analytical grade and were commercially available.

### Whole‑cell catalysis [33]

In the air supplied bioreactor (AS-BR) system, the whole-cell catalysis was conducted in a 3.0-L open-bioreactor (New Brunswick Gelligen 115) using 10 g/L *G. oxydans* containing 1 L of culture medium composed of 20 g/L biomass-based butanol, 10 g/L yeast extract, 2 g/L sorbitol, 0.5 g/L MgSO_4_, 1 g/L KH_2_PO_4_, 2 g/L K_2_HPO_4_ and 5 g/L (NH_4_)_2_SO_4_. AS-BR was fed with air at a ventilation rate of 3 vvm, which was filtered and sterilized using a 0.22 μm microfiltration membrane. Moreover, the whole-cell catalysis in an AS-BR was performed under the conditions of 500 rpm and 30 °C.

**Fig. 7:**
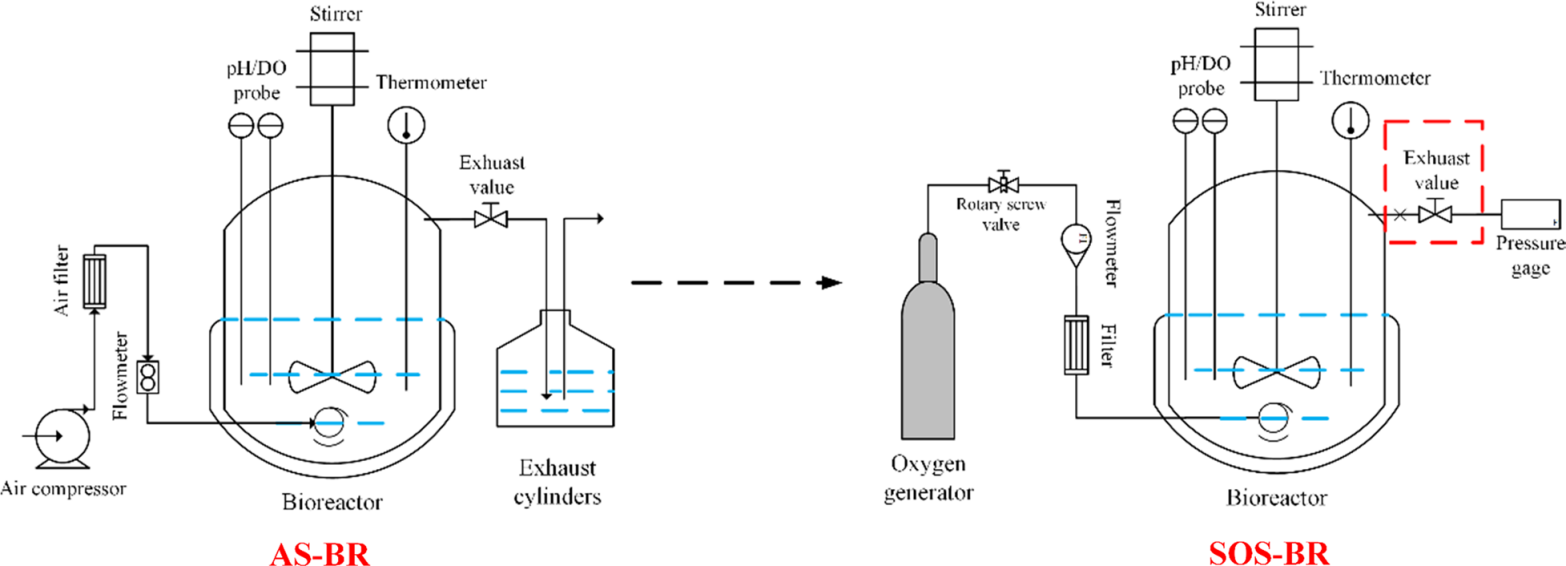
Two bioreactor operation models with different ventilation forms: AS-BR and SOS-BR

In the sealed oxygen-supplied bioreactor (SOS-BR), due to no waste gas being generated in whole-cell catalysis, the exhaust gas valve was closed and oxygen gas (Purity ≥ 99.9%) was automatically added to the stirred culture broth at an inlet pressure of 0.01–0.02 MPa. The remaining conditions, including broth medium composition and catalytic conditions, were the same as described for the AS-BR. AS-BR and SOS-BR are shown in Fig. 7 [34].

All cell-recycling technology experiments were conducted in the SOS-BR. The duration of BA production and sorbitol rejuvenation were 8 h and 4 h, respectively. After each catalytic round, the catalysis broth and *G. oxydans* cells were separated by centrifugation at 8000 rpm for 5–10 min by tubular bowl centrifuge. Subsequently, the pelleted cells were reloaded with 1 L of fresh medium containing 20 g/L biomass-based butanol or 50 g/L sorbitol.

### Volatilization rate test

Volatilization rate tests were conducted in an AS-BR without *G. oxydans* and other chemicals. In the AS-BR, an initial biomass-based butanol concentration of 100 g/L was volatilized at a ventilation capacity of 3 vvm at 30 °C and with agitation at 500 rpm. In the process of volatilization, samples were taken every 1–2 h and analyzed by chromatography.

### Analytical methods

The dissolved oxygen (DO) level was detected using a dissolved oxygen electrode (605-ISM, Mettler Toledo, USA) installed in the bottom half of the reactor. The dissolved oxygen probe was calibrated and tested before vessel insertion. The DO level was set to 100% in pure water at 25 °C with about 1.26 mmol/L oxygen concentration. Based on this method, the actual oxygen concentration can be converted by multiplying the DO level by 1.26 mmol/L.

The concentration of biomass-based butanol, BA, sorbitol and sorbose were measured by high performance liquid chromatography (HPLC) (Agilent 1260) equipped with an Aminex Bio-Rad HPX-87H column and 5 mM H_2_SO_4_ was employed as the mobile phase at 0.6 mL/min. BA yield (%) was calculated according to Eq. (2) as shown:

2 $$ {\text{BA yield }}\left( \% \right) = \frac{\text{Total final concentration of BA obtained}}{\text{Total butanol concentration}} \times 0.841, $$

where the value 0.841 is a constant, which is the molar mass ratio of biomass-based butanol to BA. The productivity was obtained by differential calculations based on the varying trends in BA concentration.

## Data Availability

All data generated and analyzed in this study are included in this published article.

## Abbreviations

BA: Butyric acid
*G. oxydans*: *Gluconobacter oxydans*
AS-BR: Air-supplied bioreactor
SOS-BR: Sealed-oxygen supply bioreactor

## References

[1] Van Immerseel F, Boyen F, Gantois I, Timbermont L, Bohez L, Pasmans F (2005). Supplementation of coated butyric acid in the feed reduces colonization and shedding of Salmonella in poultry. Poultry Sci..

[2] Canani RB, Terrin G, Cirillo P, Castaldo G, Salvatore F, Cardillo G (2004). Butyrate as an effective treatment of congenital chloride diarrhea. Gastroenterology.

[3] Fernandez-Rubio C, Ordonez C, Abad-Gonzalez J, Garcia-Gallego A, Honrubia MP, Mallo JJ (2009). Butyric acid-based feed additives help protect broiler chickens from *Salmonella enteritidis* infection. Poultry Sci..

[4] Browning M, Wallace DB, Dawson C, Alm SR, Amador JA (2006). Potential of butyric acid for control of soil-borne fungal pathogens and nematodes affecting strawberries. Soil Biol Biochem.

[5] Zigová J, Turdík E (2000). Advances in biotechnological production of butyric acid. J Ind Microbiol Biotechnol..

[6] Yu LH, Wu Z, Zhang L, Cheung CM, Yang S (2002). Production of carboxylic acids from hydrolyzed corn meal by immobilized cell fermentation in a fibrous-bed bioreactor. Bioresour Technol..

[7] Xiao ZP, Chu C, Teng B, Liu LJ, Wang B, Tao WJ, Yang ST, Wang MQ (2018). Production of butyric acid from acid hydrolysate of corn husk in fermentation by *Clostridium tyrobutyricum*: kinetics and process economic analysis. Biotechnol Biofuels.

[8] Fu H, Yu L, Lin M, Wang J, Yang ST (2016). Metabolic engineering of *Clostridium tyrobutyricum* for enhanced butyric acid production from glucose and xylose. Metab Eng.

[9] Vandak D, Telgarsky M, Turdik E (1995). Influence of growth factor supplements on butyric acid production from sucrose by *Clostridium butyricum*. Folia Microbiol.

[10] Wu Z, Yang ST (2003). Extractive fermentation for butyric acid production from glucose by *Clostridium tyrobutyricum*. Biotechnol Bioeng.

[11] Zhang CH, Ma Y, Yang F, Liu W, Zhang Y (2009). Optimization of medium composition for butyric acid production by *Clostridium thermobutyricum* using response surface methodology. Bioresour Technol..

[12] Evans PJ, Wang HY (1990). Effects of extractive fermentation on butyric acid production by *Clostridium acetobutylicum*. Appl Microbiol Biotechnol..

[13] Liu X, Zhu Y, Yang S (2006). Butyric acid and hydrogen production by *Clostridium tyrobutyricum* ATCC 25755 and mutants. Enzyme Microb Technol..

[14] Rephaeli A, Zhuk R, Nudelman A (2000). Prodrugs of butyric acid from bench to bedside: synthetic design, mechanisms of action, and clinical applications. Drug Dev Res..

[15] Hua X, Cao R, Zhou X, Xu Y (2018). Integrated process for scalable bioproduction of glycolic acid from cell catalysis of ethylene glycol. Bioresour Technol..

[16] Zhou X, Hua X, Xuelian Z, Xu Y, Zhang W (2019). Continuous co-production of biomass and bio-oxidized metabolite (sorbose) using *Gluconobacter oxydans* in a high-oxygen tension bioreactor. Bioresour Technol..

[17] Zhang H, Liu G, Zhang J, Bao J (2016). Fermentative production of high titer gluconic and xylonic acids from corn stover feedstock by *Gluconobacter oxydans* and techno-economic analysis. Bioresour Technol..

[18] Peters BR, Mientus M, Kostner D, Daniel R, Liebl W, Ehrenreich A (2017). Expression of membrane-bound dehydrogenases from a mother of vinegar metagenome in *Gluconobacter oxydans*. Appl Microbiol Biotechnol..

[19] Mientus M, Kostner D, Peters BR, Liebl W, Ehrenreich A (2017). Characterization of membrane-bound dehydrogenases of *Gluconobacter oxydans* 621H using a new system for their functional expression. Appl Microbiol Biotechnol..

[20] Meyer M, Schweiger P, Deppenmeier U (2013). Effects of membrane-bound glucose dehydrogenase overproduction on the respiratory chain of *Gluconobacter oxydans*. Appl Microbiol Biotechnol..

[21] Zhou X, Zhou X, Xu Y, Yu S (2016). Improving the production yield and productivity of 1,3-dihydroxyacetone from glycerol fermentation using *Gluconobacter oxydans* NL71 in a compressed oxygen supply-sealed and stirred tank reactor (COS-SSTR). Bioprocess Biosyst Eng..

[22] Zhou X, Zhou X, Huang L, Cao R, Xu Y (2017). Efficient coproduction of gluconic acid and xylonic acid from lignocellulosic hydrolysate by Zn(II)-selective inhibition on whole-cell catalysis by *Gluconobacter oxydans*. Bioresour Technol..

[23] Gupta A, Singh VK, Qazi GN, Kumar A (2001). *Gluconobacter oxydans*: its biotechnological applications. J Mol Microbiol Biotechnol.

[24] Prust C, Hoffmeister M, Liesegang H, Wiezer A, Fricke WF, Ehrenreich A (2005). Complete genome sequence of the acetic acid bacterium *Gluconobacter oxydans*. Nat Biotechnol.

[25] Hua X, Rou C, Zhou X, Xu Y (2018). One-step continuous/semi-continuous whole-cell catalysis production of glycolic acid by a combining bioprocess with in situ cell recycling and electrodialysis. Bioresour Technol..

[26] Zhu Y, Yang ST (2010). Adaptation of *Clostridium tyrobutyricum* for enhanced tolerance to butyric acid in a fibrous-bed bioreactor. Biotechnol Progr..

[27] Ling J, Wang J, Liang S, Wang X, Xu Z (2009). Butyric acid fermentation in a fibrous bed bioreactor with immobilized *Clostridium tyrobutyricum* from cane molasses. Bioresour Technol..

[28] Fayolle F, Marchal R, Ballerini D (1990). Effect of controlled substrate feeding on butyric acid production by *Clostridium tyrobutyricum*. J Ind Microbiol Biotechnol..

[29] Michelsavin D, Marchal R, Vandecasteele J (1990). Control of the selectivity of butyric acid production and improvement of fermentation performance with *Clostridium tyrobutyricum*. Appl Microbiol Biotechnol.

[30] Zigová J, Šturdík E, Vandák D, Schlosser Š (1999). Butyric acid production by *Clostridium butyricum* with integrated extraction and pertraction. Process Biochem..

[31] Hölscher T, Schleyer U, Merfort M, Bringer-Meyer S, Görisch H, Sahm H (2008). Glucose oxidation and PQQ-dependent dehydrogenases in *Gluconobacter oxydans*. J Mol Microb Biotechnol..

[32] Voss J, Ehrenreich A, Liebl W (2010). Characterization and inactivation of the membrane-bound polyol dehydrogenase in *Gluconobacter oxydans* DSM 7145 reveals a role in meso-erythritol oxidation. Microbiology.

[33] Zhou X, Zhou X, Xu Y (2017). Improvement of fermentation performance of *Gluconobacter oxydans* by combination of enhanced oxygen mass transfer in compressed-oxygen-supplied sealed system and cell-recycle technique. Bioresour Technol..

[34] Hua X, Zhou X, Du G, Xu Y (2020). Resolving the formidable barrier of oxygen transferring rate (OTR) in ultrahigh-titer bioconversion/biocatalysis by a sealed-oxygen supply biotechnology (SOS). Biotechnol Biofuels.

